# How Structural Subtleties Modulate Excited‐State Lifetimes in Cyclometalated Cobalt(III) Complexes: Nonadiabatic Molecular Dynamics Study

**DOI:** 10.1002/jcc.70453

**Published:** 2026-07-20

**Authors:** Hamada Rezk, Oliver Kühn, Olga S. Bokareva

**Affiliations:** ^1^ Institute of Physics, University of Rostock Rostock Germany; ^2^ Leibniz Institute for Catalysis (LIKAT) Rostock Germany; ^3^ Institute of Chemistry, University of Rostock Rostock Germany

## Abstract

Ultrafast excited‐state dynamics of two Co(III) complexes, bearing different alkyl groups (**Co1**: Methyl, **Co2**: Ethyl) at the nitrogen of the imidazole ligand, are investigated using trajectory surface hopping based on a linear vibronic coupling model. Despite nearly identical MLCT absorption spectra, experimental singlet lifetimes differ markedly (1.45 vs. 4.3 ps). Relaxation occurs via rapid internal conversion within a strongly vibronically coupled singlet manifold, accompanied by intersystem crossing to long‐lived 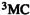 states. The prolonged lifetime of **Co2** originates from enhanced vibronic coupling and larger structural distortions, which trap population in the singlet manifold rather than from spin–orbit effects. Minimum‐energy crossing points reveal low barriers for nonradiative decay, rationalizing emission quenching. These results demonstrate how subtle structural differences, by modulating vibronic coupling, can substantially influence excited‐state lifetimes in these complexes.

## Graphical abstract

Nonadiabatic trajectory simulations unravel the origin of different relaxation times scales observed for two Co(III) complexes, bearing different alkyl groups at their ligands.

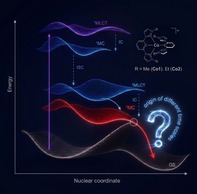

## 1 Introduction

Transition‐metal complexes, particularly those based on noble metals, are renowned for their rich photochemical and photophysical behavior, which arises largely from their long‐lived electronically excited states formed upon photoexcitation [1]. These extended lifetimes enable the efficient utilization of excess energy either directly—through luminescence in applications such as biomedical imaging and therapy [2]—or indirectly, by driving charge separation processes in solar energy conversion technologies [3].

Achieving comparable functionality in first‐row 3*d* transition‐metal complexes remains a significant challenge and continues to motivate extensive research efforts [4]. This limitation primarily stems from the relatively weak ligand‐field splitting in 3*d* metals compared to their heavier 4*d* and 5*d* counterparts. As a consequence, low‐lying metal‐centered (MC) states often become accessible and act as efficient nonradiative decay pathways, substantially shortening excited‐state lifetimes. Overcoming these limitations is particularly attractive given the greater earth abundance and lower cost of 3*d* transition metals, which offer a pathway toward more sustainable and economically viable technologies.

Among first‐row transition‐metal complexes with partially filled d orbitals, six‐coordinate chromium(III) complexes have attracted considerable attention [5]. In octahedral $d^{3}$ systems, the lowest spin‐flip metal‐centered (MC) excited state involves only minimal structural distortion relative to the ground state, which is a key factor underlying their remarkably long‐lived luminescence [6, 7]. In contrast, metal‐to‐ligand charge‐transfer (MLCT) excited states in nickel(II) ($3d^{8}$) [8, 9] and iron(II) ($3d^{6}$) [10] complexes undergo ultrafast deactivation, severely limiting their applicability in processes that rely on long‐lived excited states. This behavior stands in stark contrast to that of their second‐ and third‐row $d^{8}$ and $d^{6}$ counterparts, where long‐lived MLCT states play a central role in photophysical and photochemical processes [11, 12]. Photoactive iron(II) complexes have been extensively investigated over several decades, with particular emphasis on elucidating their excited‐state deactivation pathways [13, 14, 15, 16, 17], extending MLCT lifetimes [18], and exploring their applications in photoredox catalysis [19, 20].

In contrast, the photophysics and photochemistry of isoelectronic cobalt(III) complexes have received comparatively less attention [21, 22, 23]. The higher oxidation state of cobalt(III) relative to iron(II) results in a stronger ligand field, which helps suppress nonradiative relaxation via distorted MC excited states [24]. A particularly striking example is provided by the hexacyanocobaltate(III) complex, [Co(CN)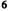]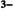, in which the lowest triplet MC state is raised sufficiently in energy that luminescence can effectively compete as a deactivation pathway [25].

Recently, Krishna et al. [26] reported a family of cobalt(III) complexes, [Co(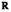
ImP)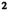][PF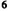] (H
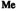
ImP = 1,$1^{'}$‐(1,3‐phenylene)bis(3‐methyl‐1‐imidazole‐2‐ylidene)), in which the donor strength of the ImP ligand is tuned via the alkyl substituent (R = Me, Et, *i*Pr, and *n*Bu). The underlying rationale is that increasing σ‐donor strength at the imidazole nitrogen modulates the energetic positions of states with metal‐centered (MC) character, thereby reshaping the excited‐state landscape and influencing the time scales of internal conversion (IC) and intersystem crossing (ISC). However, experimental absorption and emission spectra show little dependence on the ligand substituent. Notably, phosphorescence from the ^3^MC state is observed only at 77 K, while none of the complexes are emissive at room temperature.

Analysis of the room‐temperature transient absorption data revealed three characteristic time scales for all complexes, with tentative assignments based on analogy to Fe(II) systems. The shortest component ($\tau_{1}$), in the range of 1–4 ps, was attributed to ISC from the ^1^MLCT to the ^3^MLCT state. The longest component ($\tau_{3}$), spanning 1–5 ns, was assigned to ground‐state recovery from the ^3^MC state. The most pronounced variation among the complexes was observed for the intermediate time scale ($\tau_{2}$), which ranged from 7 to 11 ps for most compounds but increased to 162 ps for the Et‐substituted complex. This component was assigned to IC from the ^3^MLCT to the ^3^MC state.

However, while the experimental study clearly demonstrates the sensitivity of excited‐state lifetimes to ligand functionalization, the mechanistic origins of this behavior remain unresolved. In particular, it is unclear how subtle electronic and steric perturbations introduced by alkyl substitution reshape the topology of the potential energy surfaces, influence nonadiabatic couplings, and affect vibrational energy redistribution governing excited‐state decay. The coexistence of nearly identical absorption spectra with markedly different excited‐state dynamics highlights the need for a combined quantum‐chemical and dynamical investigation.

From a theoretical perspective, transition‐metal complexes pose significant challenges due to the interplay of electronic degeneracies, strong mixing of states with different character, spin–orbit interactions, nonadiabatic couplings, and a high density of states [27]. While density functional theory (DFT) often provides a practical balance between accuracy and computational cost—particularly when employing optimally tuned range‐separated functionals [28]—it cannot be treated as a black‐box approach for such systems [29, 30]. More recently, many‐body Green's function methods have demonstrated improved robustness for first‐row transition‐metal complexes [31].

For the description of excited‐state dynamics, trajectory surface hopping (TSH) [32, 33] is widely employed [27, 34], but its application requires on‐the‐fly evaluation of potential energy surfaces, which is often computationally prohibitive for transition‐metal systems. As an efficient alternative, vibronic coupling Hamiltonians have been successfully introduced in TSH simulations [35, 36]. Within this framework, the Hamiltonian is constructed in a diabatic representation using normal‐mode coordinates and harmonic potentials, where interstate couplings and shifts in equilibrium geometries are obtained from low‐order Taylor expansions. Retaining only linear terms leads to the linear vibronic coupling (LVC) model [37, 38, 39]. The combination of LVC models with TSH has enabled the investigation of the photophysics of a wide range of transition‐metal complexes, including solvent effects [30, 40, 41, 42, 43]. Furthermore, the relatively simple structure of the vibronic Hamiltonian also permits numerically exact quantum dynamics simulations [44, 45].

**FIGURE 1:**
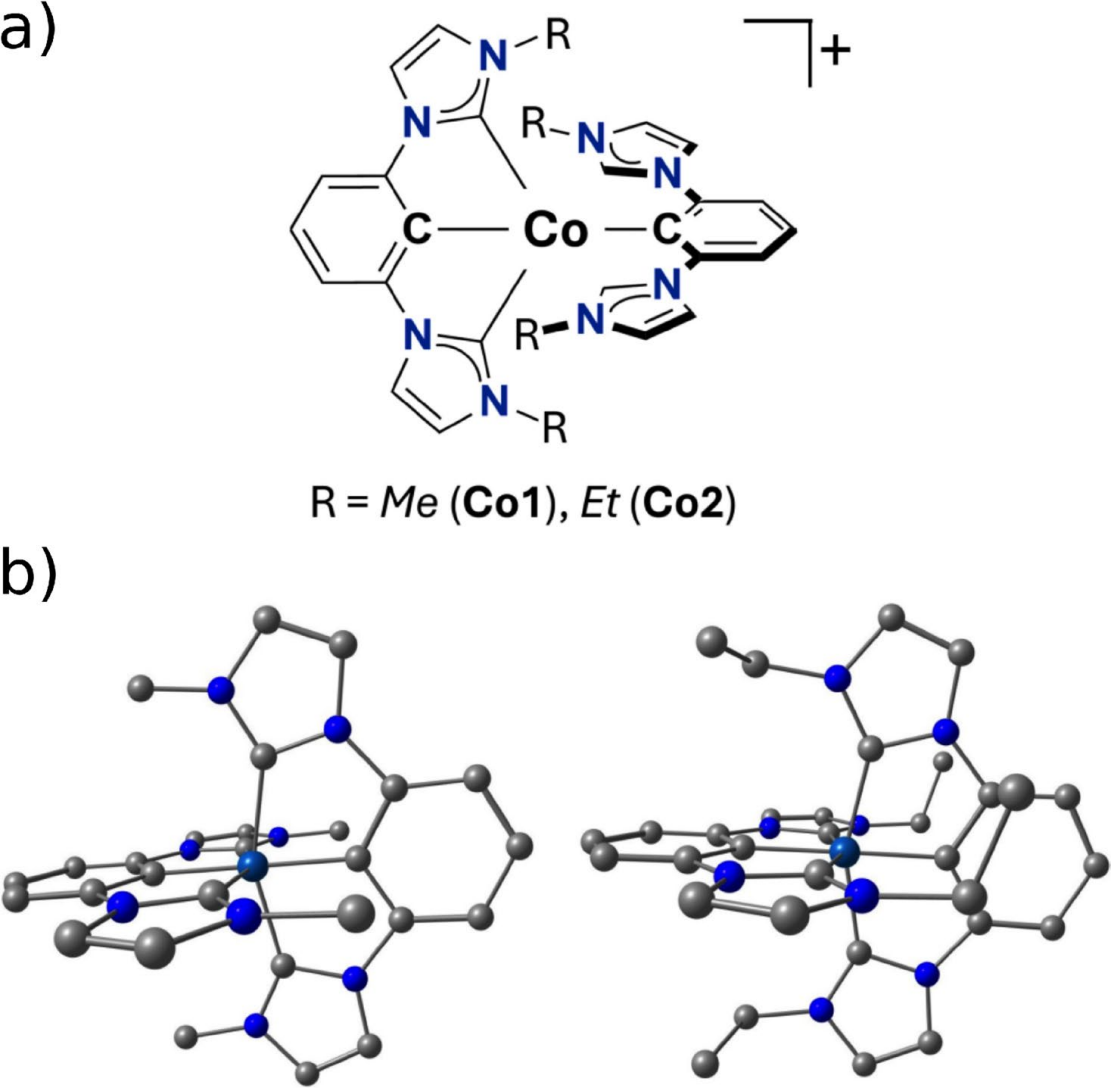
(a) [Co(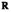
ImP)]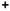 (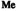
ImP = 1,1′‐(1,3‐phenylene)bis(3‐methyl‐1‐imidazole‐2‐ylidene)) with R = Me (**Co1**) and Et (**Co2**). (b) Optimized structures of **Co1** (left) and **Co2** (right) in acetonitrile; hydrogen atoms are omitted for clarity.

In this work, we combine electronic‐structure calculations with nonadiabatic dynamics simulations based on a LVC Hamiltonian to elucidate the excited‐state relaxation pathways in cobalt(III) complexes. We focus on two representative systems, R = Me (**Co1**) and R = Et (**Co2**), which exhibit markedly different transient absorption time scales ($\tau_{1}=1.2/4.3$ ps, $\tau_{2}=7/162$ ps, and $\tau_{3}=1/2$ ns for **Co1**/**Co2**, respectively), despite their close structural similarity (Figure 1). By directly comparing these complexes, we aim to uncover how subtle ligand modifications control the interplay between MLCT and MC states, and ultimately determine the observed differences in excited‐state lifetimes. We begin by outlining the theoretical framework for excited‐state dynamics, followed by the construction and analysis of the linear vibronic coupling models and their associated dynamics simulations, and conclude with a discussion of the mechanistic insights obtained.

## 2 Theoretical and Computational Methods

Nonadiabatic dynamics simulations were performed using TSH within the framework of the LVC model. In this approach, the Hamiltonian is formulated in a diabatic representation as [37].

(1) $$H^{\left(\text{diab}\right)}=\sum_{\mathrm{mn}}\left[\delta_{\mathrm{mn}}H_{m}\left(Q\right)+\left(1-\delta_{\mathrm{mn}}\right)V_{\mathrm{mn}}\left(Q\right)\right]\left|m\right\rangle \left\langle n\right|,$$

where $\left|m\right\rangle$ denote diabatic electronic states defined at fixed nuclear coordinates, and $Q=\left\{Q_{\xi }\right\}$ are the (harmonic) normal‐mode coordinates of the electronic ground state.

The diagonal elements of the Hamiltonian are expressed as

(2) $$H_{m}\left(Q\right)=E_{m}+\frac{1}{2}\sum_{\xi }\hbar \omega_{\xi }\left(\frac{\partial^{2}}{\partial Q_{\xi }^{2}}+Q_{\xi }^{2}\right)+\sum_{\xi }\kappa_{m,\xi }Q_{\xi },$$

where $E_{m}$ are vertical excitation energies, $\omega_{\xi }$ are normal‐mode frequencies, and $\kappa_{m,\xi }$ are linear vibronic coupling constants corresponding to first‐order derivatives of the potential energy surfaces (PESs). The off‐diagonal elements (m≠n) are approximated as

(3) $$V_{\mathrm{mn}}\left(Q\right)=V_{\mathrm{mn}}^{\left(\mathrm{SOC}\right)}+\sum_{\xi }\lambda_{\mathrm{mn},\xi }Q_{\xi },$$

where $V_{\mathrm{mn}}^{\left(\mathrm{SOC}\right)}$ are spin–orbit coupling (SOC) matrix elements and $\lambda_{\mathrm{mn},\xi }$ are interstate vibronic coupling constants.

The LVC parameters ($E_{m}$, $\kappa_{m,\xi }$, and $\lambda_{\mathrm{mn},\xi }$) were determined from quantum chemical calculations at the Franck–Condon geometry and along selected normal‐mode displacements.

Electronic structure calculations were performed using linear‐response time‐dependent density functional theory (TD‐DFT) with the optimally tuned, long‐range corrected LC‐BLYP functional, which has been shown to provide an accurate description of charge‐transfer states in transition‐metal complexes [28, 30]. A mixed basis set was employed, using def2‐TZVP for cobalt and 6‐311G(d,p) for all other atoms. The functional parameters were tuned via the ΔSCF approach [46], yielding α=0 and a range‐separation parameter of $0.15\;{\text{Bohr}}^{-1}$ (see Supporting Information, Section S1).

Solvent effects (acetonitrile) were included using the polarizable continuum model (PCM) [47]. Frequency calculations confirmed the absence of imaginary modes for the stationary geometries (see Supporting Information, Section S2). Excited states were computed within the Tamm–Dancoff approximation (TDA). Minimum energy crossing points (MECPs) between the ^3^MC and ^1^GS states were obtained by optimizing the crossing geometry starting from the ^3^MC minimum.

The vibronic coupling constants $\kappa_{m,\xi }$ and $\lambda_{\mathrm{mn},\xi }$ were obtained numerically using finite displacements ($\delta Q_{\xi }=0.05$) and wave function overlaps between displaced geometries [42, 48, 49]. Wave function overlaps were computed with a determinant inclusion threshold of 0.9999. The LVC Hamiltonian included the lowest thirteen singlet and triplet states and was constructed using the SHARC 3.1 package [50]. Spin–orbit couplings were calculated at the ground‐state equilibrium geometry using the Breit–Pauli operator [27]. All electronic‐structure calculations were carried out with ORCA 5.0 [51, 52], and excited‐state analyses were performed using the TheoDORE package [53].

TSH simulations were performed using SHARC 3.1 with Tully's fewest‐switches algorithm. A three‐step propagator including transformations between adiabatic/spin‐diabatic and diagonal representations, as well as local diabatization, was employed. This local diabatization is based on overlap‐driven state tracking within the surface‐hopping propagation, enabling a state‐character‐based mapping of adiabatic states through the continuous evolution of their electronic character along the trajectories. Nuclear and electronic time steps of 0.5 fs and 0.005 fs were used, respectively. Gradients were computed in the adiabatic/spin‐diabatic basis and transformed into the diagonal representation with appropriate nonadiabatic coupling corrections. Decoherence effects were included using the energy‐based correction scheme with a parameter of 0.1 a.u. [54].

Initial conditions were generated from a harmonic Wigner distribution around the optimized ground‐state geometry. A total of 500 initial conditions were sampled and filtered within an energy window from 3.6 to about 4.0 eV, that is, covering the lower peak of the theoretical spectrum. Approximately 170 initial conditions were selected and propagated for 1 ps. Initial states correspond to excitation into the ^3^MLCT manifold, and oscillator strengths were used for statistical weighting. Surface hopping probabilities were computed using wave function overlaps [55], and kinetic energy was adjusted via velocity rescaling upon hops.

During preliminary simulations, instabilities associated with low‐frequency vibrational modes were observed, leading to artificial energy spreading and nonphysical population transfer. Such effects are known to arise from numerical noise and limitations of the harmonic approximation in LVC models [35, 42, 56, 57]. To mitigate these issues, the five lowest‐frequency modes were excluded from the LVC Hamiltonian.

Electronic state populations were analyzed to characterize the excited‐state dynamics. The role of vibrational degrees of freedom was further quantified using normal‐mode activities defined as [58].
(4)

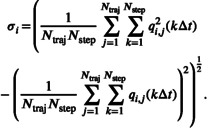




Here, $N_{\text{traj}}$ and $N_{\text{step}}$ denote the number of trajectories and time steps, respectively.

## 3 Results and Discussion

### 3.1 Assignment of Absorption Spectrum

**FIGURE 2:**
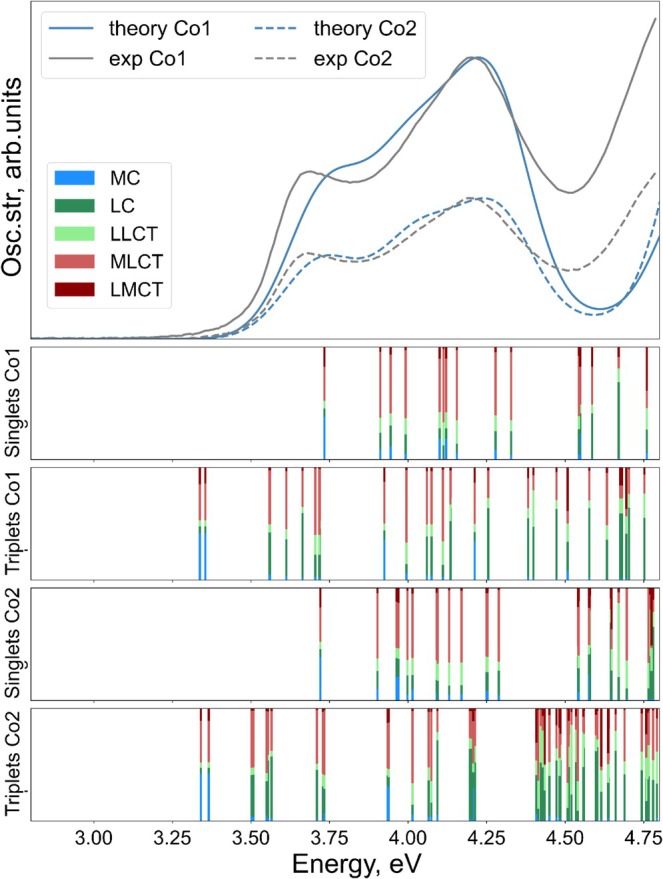
Overlay of the simulated optical absorption spectrum for **Co1** and **Co2**, in acetonitrile (blue line, LC‐BLYP) and the experimental spectrum (grey line). A Gaussian broadening with a width of 0.2 eV has been applied. Notice that experimental and theoretical spectra have been normalized separately to the maximum at about 4.2 eV.

The experimental absorption spectra of the **Co1** and **Co2** complexes are shown in Figure 2 [26]. The TD‐DFT calculated spectra, obtained using the optimally tuned LC‐BLYP functional, are in good agreement with experiment.

**FIGURE 3:**
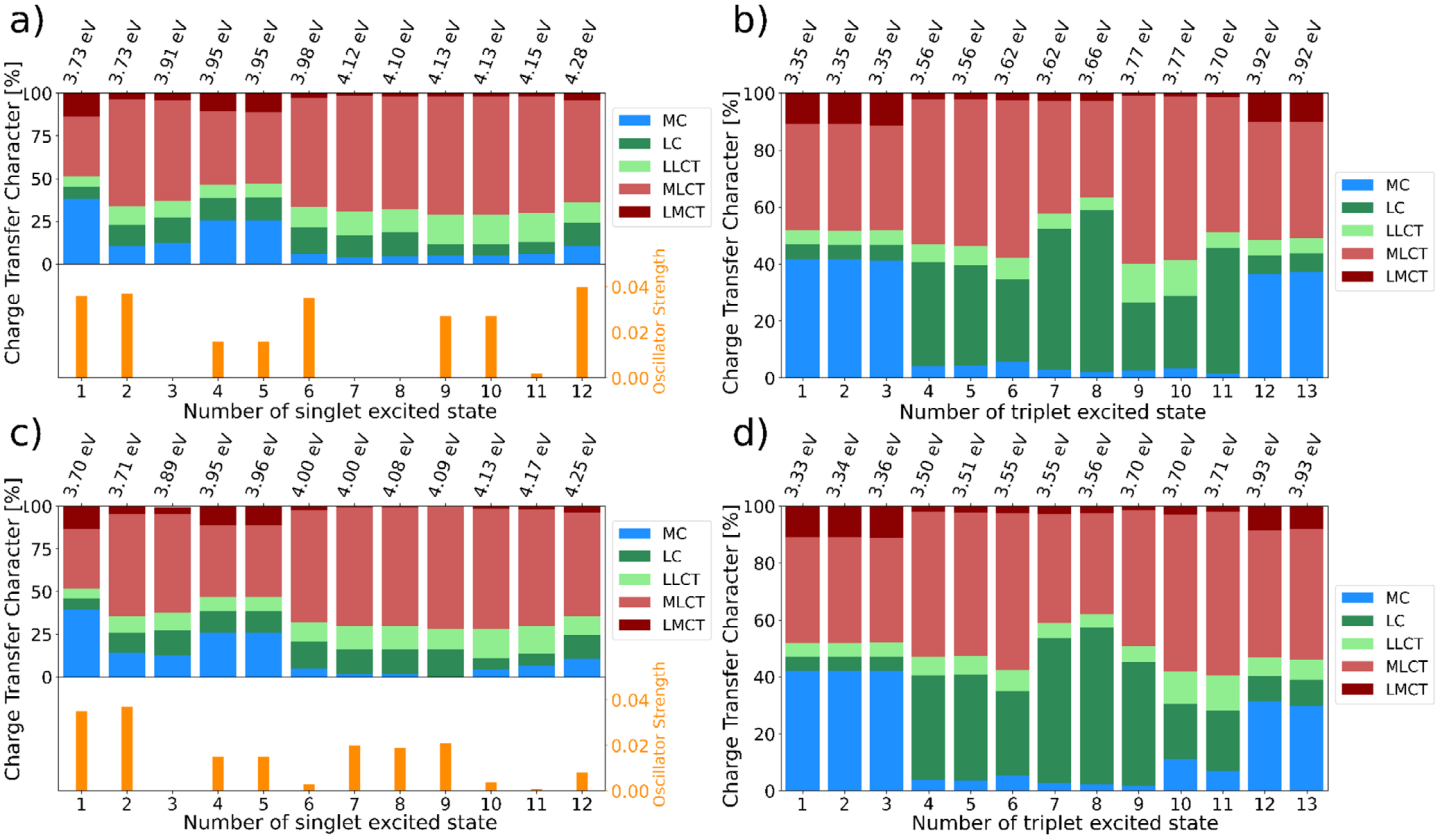
Density‐matrix analysis of the lowest singlet and triplet states included into the LVC model. (a) S
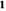
‐S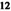, and (b) T
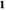
‐T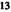 for **Co1**, (c) S
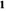
‐S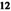, and (d) T
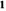
‐T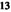 for **Co2**. Panels (a) and (c) show the oscillator strength for transitions from the ground state.

To elucidate the electronic character of the excited states, a systematic wavefunction analysis was performed by partitioning each complex into three fragments: the cobalt center and the two imidazolyl–phenylene (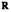
ImP) ligands. As illustrated in Figure 2 (see also Figure 3), the low‐energy region up to approximately 4.2 eV is dominated by metal‐to‐ligand charge‐transfer (MLCT) transitions, corresponding to excitations from singly and doubly occupied Co(III) d‐orbitals to ligand $\pi^{*}$‐orbitals. These transitions exhibit varying contributions from metal‐centered (MC) excitations between Co(III) d‐orbitals, as well as a small admixture of ligand‐centered (LC) character arising from covalent metal–ligand interactions.

At higher energies (above ∼4.2 eV), the excited states become increasingly mixed, comprising ligand‐to‐metal charge‐transfer (LMCT) transitions from ligand π‐orbitals to Co(III) d‐orbitals, ligand–ligand charge‐transfer (LLCT) transitions between π and $\pi^{*}$‐orbitals of different ligands, as well as additional LC contributions. Overall, both the spectral shapes and the underlying state character show only minor differences between **Co1** and **Co2**.

### 3.2 Analysis of the LVC Model

The LVC model includes the 13 lowest singlet states (the ground state, S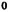, and the 12 lowest singlet excited states, S
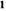
–S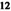) and the 13 lowest triplet states (T
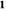
–T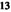) In Figure 3, we analyze the nature of these transitions in more detail. Overall, the singlet excitations of **Co1** and **Co2** do not differ much in transition energy, but there is noticeable variation in character. All transitions have a substantial MLCT character, except S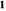 where MC dominates. The MC type is also prominent in S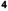 and S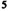. Further, there is a certain fraction of LLCT and LC participation across the whole spectrum.

Triplet states also show little variation in energies between the two systems. Within the triplet manifold, states T
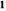
‐T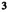, T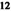, and T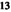 show MC character in both **Co1** and **Co2**. States T
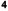
–T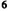, and T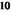 exhibit MLCT character with significant LC contributions, while T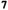 and T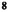 are predominantly LC with MLCT contribution. Notable differences between **Co1** and **Co2** are observed for states T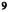 and T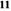. In **Co1**, T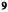 is primarily MLCT with a significant LC contribution, whereas in **Co2** it is predominantly LC with notable MLCT character. Conversely, T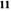 displays LC character with MLCT contribution in **Co1**, but MLCT character with significant LC contribution in **Co2**. In addition, the MC contribution to states T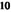 and T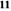 is significantly larger in **Co2** than in **Co1**.

The Et group is nominally a stronger σ‐donor than Me, that is, its primary effect is a selective stabilization of MC states. Mixing with MLCT states, however, obscures this simple view, and as already noticed, there is little variation in excitation energies between the two systems. The lowest 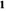
MC state is slightly lower in energy in **Co2** (3.70 eV) compared to **Co1** (3.73 eV). A similar change is observed in the triplet manifold, where the lowest 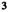
MC states are lowered by approximately 0.02 eV upon Et substitution.

Overall, these results demonstrate that alkyl substitution in [Co(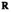
ImP)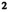] complexes has only a small effect on excitation energies, which is in accord with the almost identical absorption spectra in Figure 2. Nevertheless, Krishna et al. [26] reported that structurally related [Co(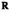
ImP)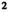] complexes exhibit markedly different excited‐state dynamics. Their origin cannot be large differences in transition energies, but more subtle electronic structure variations. Indeed, the composition of the transitions in Figure 3 is rather similar, but not identical. This observation should reflect in matrix elements such as those entering the LVC Hamiltonian.

**FIGURE 4:**
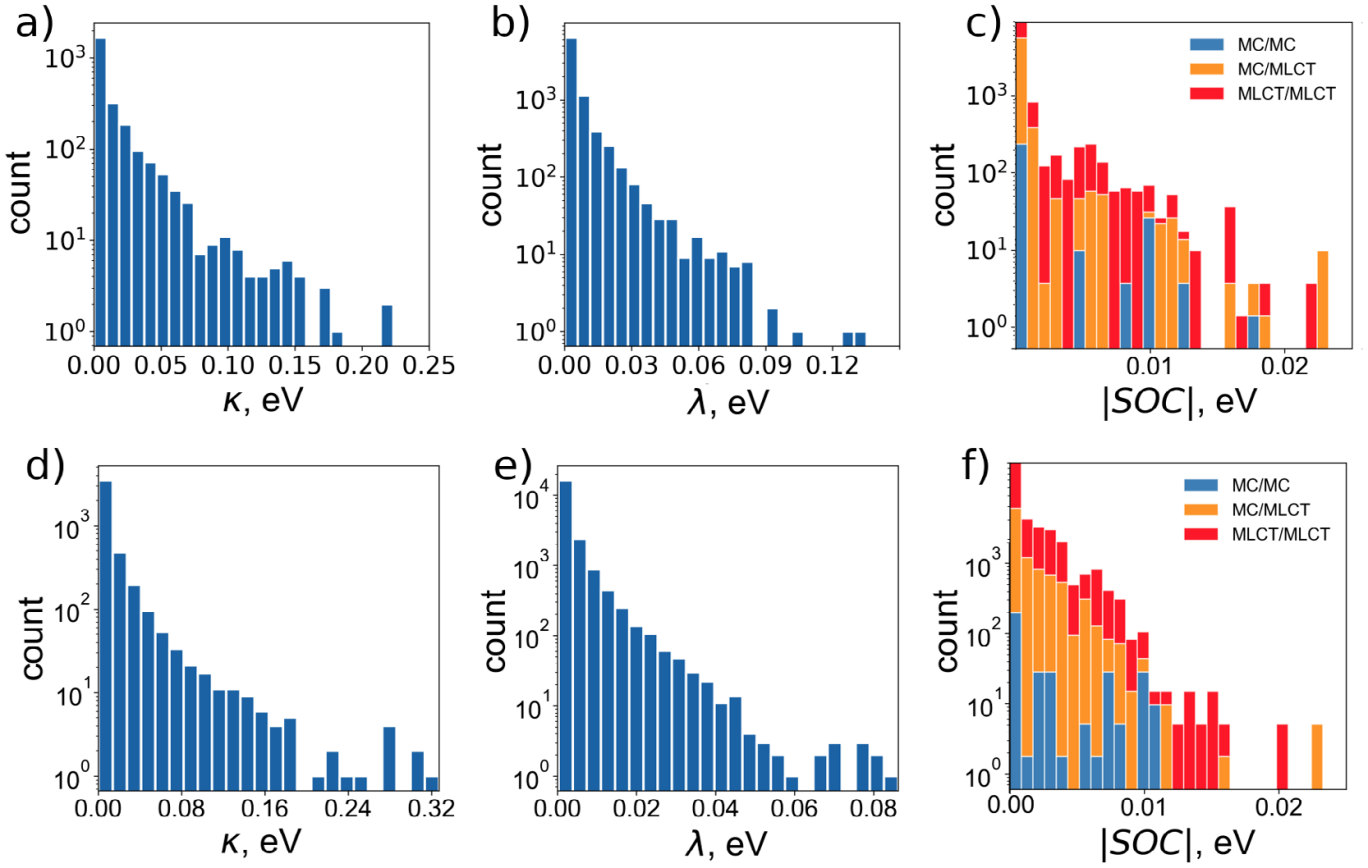
Distribution of coupling parameters in the LVC and SOC Hamiltonians. Panels (a–c) correspond to **Co1**, while panels (d–f) correspond to **Co2**. Specifically, (a,d) $\kappa =|\kappa_{m,\xi }|$ representing state‐specific vibronic couplings; (b,e) $\lambda =|\lambda_{\mathrm{mn},\xi }|$ indicating inter‐state vibronic couplings, and (c,f) $|\mathrm{SOC}|=|V_{\mathrm{mn}}^{\left(\mathrm{SOC}\right)}|$ denoting spin‐orbit coupling strengths between electronic states grouped by state character.

Figure 4 shows an analysis of the LVC parameters for **Co1** (panels a–c) and **Co2** (panels d–f). To eliminate small contributions prone to numerical noise, an energy threshold of 0.0003 eV was applied. The present model has 178 and 214 modes for **Co1** and **Co2**, respectively. In total, there are 3751 $\kappa_{m,\xi }$ terms, 16,866 $\lambda_{\mathrm{mn},\xi }$ terms, and 1800 SOC terms for **Co1**. In comparison, the LVC Hamiltonian for **Co2** contains 5434 $\kappa_{m,\xi }$ terms, 22,755 $\lambda_{\mathrm{mn},\xi }$ terms, and 1187 SOC terms.

The majority of the coupling terms are small in magnitude. For the case of **Co2**, there are more $\kappa_{m,\xi }$ terms beyond 0.2 eV, as compared with **Co1**. Concerning interstate couplings **Co1** features two $\lambda_{\mathrm{mn},\xi }$ pairs with magnitudes above 0.12 eV (coupling between T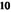 and T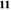 via two different modes). The SOCs are relatively small, that is, below ∼0.023 eV. In Figure 4c,f, they are classified according to which types of states are coupled. Changing the alkyl group has a profound influence on the distribution of SOCs. Above 0.02 eV, one finds in both cases SOCs between states of MC‐MLCT and MLCT‐MLCT type. Overall, however, MC‐MC type couplings are more prominent on **Co2**.

### 3.3 Population Dynamics

**FIGURE 5:**
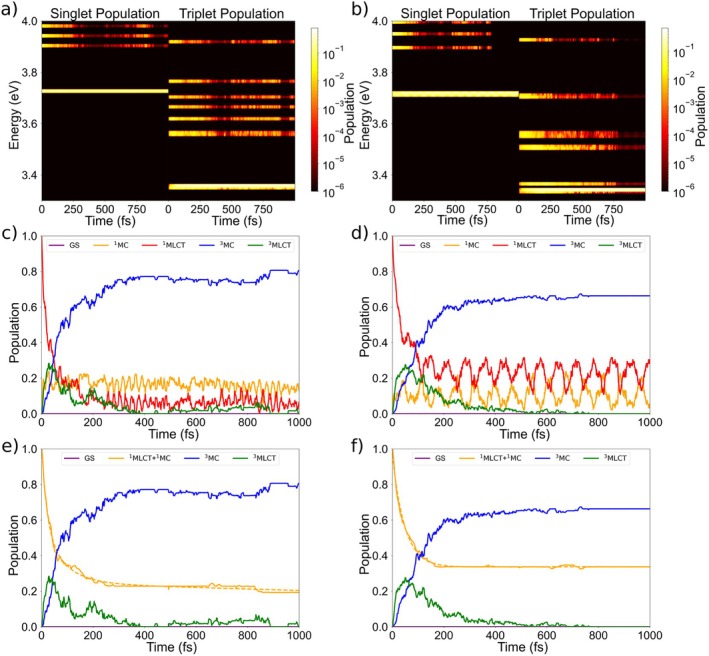
Analysis of TSH simulations for the **Co1** and **Co2** complexes. Panels (a, c, and e) correspond to **Co1**, while panels (b, d, and f) correspond to **Co2**. (a,b) Time evolution of the diabatic S
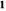
–S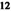 and T
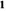
–T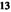 state populations, shown at their respective vertical excitation energies at FC geometry (logarithmic scale). (c,d) Diabatic state populations grouped according to excited‐state character. (e,f) Time evolution of the 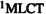, 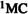, 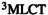, and 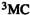 populations, fitted with exponential functions (dashed lines).

Figure 5 shows the time evolution of the diabatic populations for singlet and triplet states according to the TSH simulations for **Co1** and **Co2**. The electronic states are represented in the spin‐free adiabatic basis, where they are distinguished by their spin multiplicity (singlets and triplets) and energetic ordering. Based on the defined excitation window (up to 4 eV), trajectories were stochastically initialized in all states accessible according to oscillator strength (cf. Figure 3). Consequently, the initial population is distributed across this range.

Previous theoretical studies on excitation into near‐degenerate electronic manifolds have shown that the initial wavepacket preparation can depend on the details of the excitation process and may influence ultrafast coherent dynamics. However, in systems with dense electronic manifolds and strong vibronic and spin–orbit coupling, rapid internal relaxation typically leads to redistribution within the same low‐lying excited‐state manifold, rendering the longer‐time dynamics largely insensitive to the precise nature of the initial excitation conditions [59, 60].

As observed in Figure 5a,b, the population initially present in the higher states rapidly relaxes to the two lowest states ($S_{1}$ and $S_{2}$) within the first ~150 fs. In both systems, this initial relaxation is followed by a periodic population exchange between these two states. This quantum beating signifies the vibronic coupling between states having mixed ^1^MC and ^1^MLCT character. It is further analyzed in Section S6, Supporting Information.

Figure 5 also shows that ISC to the triplet manifold parallels the initial relaxation and proceeds afterward. Upon population of the triplet manifold, IC occurs from the 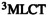 to 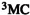 states as seen in Figure 5c,d. Figure 5e,f contains the summed populations for singlet, 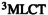 and 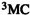 states.

Overall, the relaxation pathway can be summarized as ^1^MC + ^1^MLCT → ^3^MLCT → ^3^MC. This differs slightly from the mechanism proposed by Krishna et al. [26]. In that work, the relaxation cascade (^1^MLCT → ^3^MLCT → ^3^MC) is inferred based on analogy to the well‐known excited‐state landscape of Fe(II) complexes. However, the transient absorption components associated with the time constants $\tau_{1}$ and $\tau_{2}$ cannot be unambiguously assigned, indicating that the early‐time dynamics remain only partially resolved. In this context, the involvement of ^1^MC states is not explicitly considered. In contrast, our results reveal a significant contribution of ^1^MC character, highlighting the role of ultrafast IC within the singlet manifold prior to ISC.

To assign time constants for comparison with experiment, the decay of the singlet manifold is fitted to a biexponential function (dashed lines). In the following, the focus will be on the slow component because the initial fast decay has not been observed in the experiment and is dependent on the initial conditions of the TSH simulation. The slower decay time constants of the singlet manifold found from the present simulations are 1.45 ps for **Co1** and 4.3 ps for **Co2**. These values are in excellent agreement with the 1.2 ps and 4.3 ps reported by Krishna et al. [26]. The experimental time constants tentatively assigned to the ^3^MLCT → ^3^MC relaxation (7 and 162 ps) are beyond the time window of the present simulation. However, the authors of Ref. [26] themselves admit that unambiguous assignment of $\tau_{2}$ is hampered by the fact that the related decay‐associated spectra are significant in the UV only and, in particular, the 162 ps component is rather weak. In addition, a possible structural relaxation of the ^3^MC state is suggested. Although it appears that, according to the TSH simulations, the ^3^MLCT → ^3^MC relaxation could be much faster (the population of ^3^MLCT undergoes rapid decay within 1 ps, as shown in Figure 5e,f), a careful assignment of these longer time scales would require calculations of excited‐state absorption, in combination with going beyond the LVC model. For further discussion, see Supporting Information, Section S3.

Although quintet states often play an important role in the excited‐state dynamics of Fe(II) complexes [60], the ^3^MC geometry in the present cases is significantly different from the ground state and both ^3^MC and ^3^MLCT excited states, as reflected by the pronounced elongation of the Co—C bonds and strong distortion of the coordination sphere (see the Supporting Information, Table S1). In particular, both axial and equatorial Co—C bonds are substantially elongated compared to the ground state, accompanied by a marked deviation from linearity at the metal center. This results in a strongly distorted octahedral geometry, in contrast to the only moderately perturbed ^3^MC structure and the near‐ground‐state‐like ^3^MLCT state. In addition, the ^3^MC state lies significantly higher in energy than the corresponding triplet states (see Table S1) in agreement with results for other Cobalt(III) compounds [21, 23]. Overall, the ^3^MC configuration is both energetically and structurally removed from the accessible excited‐state region, indicating that it is not involved in the dominant deactivation pathway of the present Co(III) complexes.

In a first step to rationalize the longer lifetime of the coupled singlet manifold ($\tau_{1}$) observed for **Co2** relative to **Co1**, we consider the bare SOC problem, focusing on the transitions out of the states S_1_ and S_2_ into the triplet manifold. Treating each pair of S‐T states as a coupled two‐level system, an initially prepared singlet state will perform periodic oscillations with the transition amplitude $P_{S\to T}=\left(4{\left|V_{\mathrm{SOC}}\right|}^{2}/{\left(\hbar \Omega \right)}^{2}\sin^{2}\left(\Omega t/2\right)\right)$ with $\hbar \Omega =\sqrt{{\left(E_{S}-E_{T}\right)}^{2}+4{\left|V_{\mathrm{SOC}}\right|}^{2}}$. Where $V_{\mathrm{SOC}}$ is the SOC matrix element between the singlet and triplet states and Ω is the angular frequency of the singlet–triplet oscillation [39]. Hence at T=π/Ω the triplet state is populated by an amount of $4{\left|V_{\mathrm{SOC}}\right|}^{2}/{\left(\hbar \Omega \right)}^{2}$, depending on the detuning and the coupling. For **Co1**, we find that the triplet states close to resonance have low fractional populations of 0.04 (T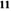). The related transfer times are about 55 fs. For **Co2**, there is a resonant triplet state (S_1_‐T_10_), that is, the population after half a period equals one, but the associated transfer time is about 900 fs. A detailed analysis is given in the Supporting Information, Section S5. Clearly, this analysis of the bare SOC problem cannot account for the observed difference in time scales.

**FIGURE 6:**
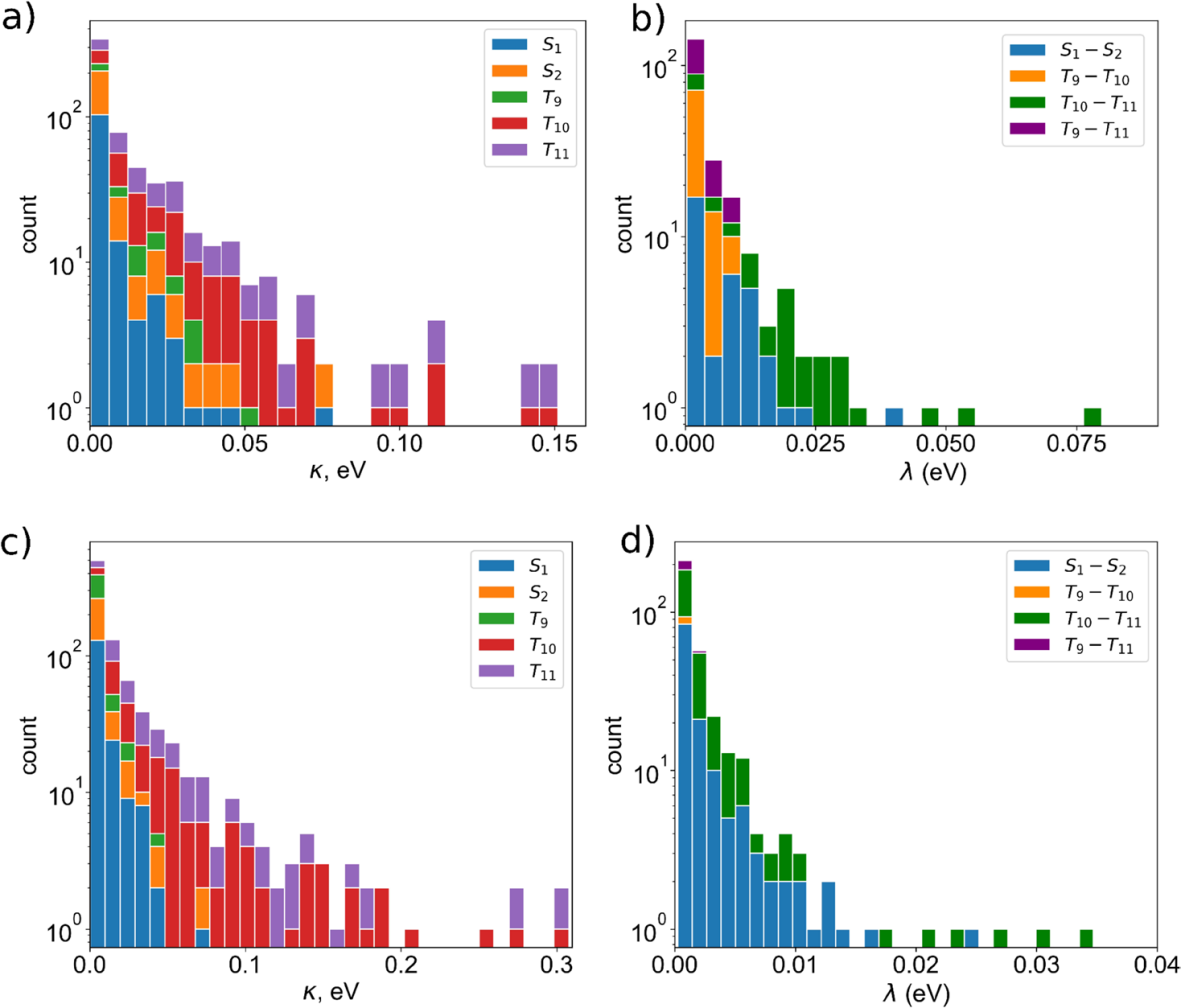
Distribution of coupling parameters in the LVC Hamiltonian for the lowest singlet and respective resonant triplet states as indicated. Panels (a, b) correspond to **Co1**, while panels (c, d) correspond to **Co2**.

The origin of the difference becomes more transparent by inspecting the LVC parameters governing the initially populated singlet states and their coupling to the low‐lying triplet manifold. In both complexes, the dominant singlet states involved in the early‐time dynamics are S_1_ and S_2_, while the relevant triplet acceptor states are T
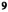
–T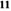. The distributions of the corresponding κ, λ, and spin–orbit coupling (SOC) parameters are summarized in Figure 6.

For **Co1**, the κ values remain within approximately 0.15eV, with the largest displacements associated primarily with the triplet states, particularly T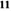. In contrast, **Co2** exhibits a broader distribution of κ values, extending up to approximately 0.3eV. It is important to emphasize that having a larger spread of displacements between singlet and triplet states could lead to effectively higher barriers along normal mode coordinates connecting singlet and triplet states and thus slower dynamics.

Concerning the vibronic coupling parameters λ, we notice that in the singlet manifold the span of mode couplings is broader for **Co2** as compared to **Co1**. This finding gives further support for the notion that in **Co2** structural distortion in the S_1_/S_2_ states is more prominent. Interestingly, in the triplet manifold, there are a few large λ values for **Co1**. This fact could favor larger distortions after ISC. As a preliminary conclusion, we note that according to the coupling parameters of the LVC + SOC Hamiltonian, it is not the bare SOC that is responsible for the different singlet‐triplet transition rates, but the distinct vibronic coupling of the two systems.

### 3.4 Vibrational Dynamics

**FIGURE 7:**
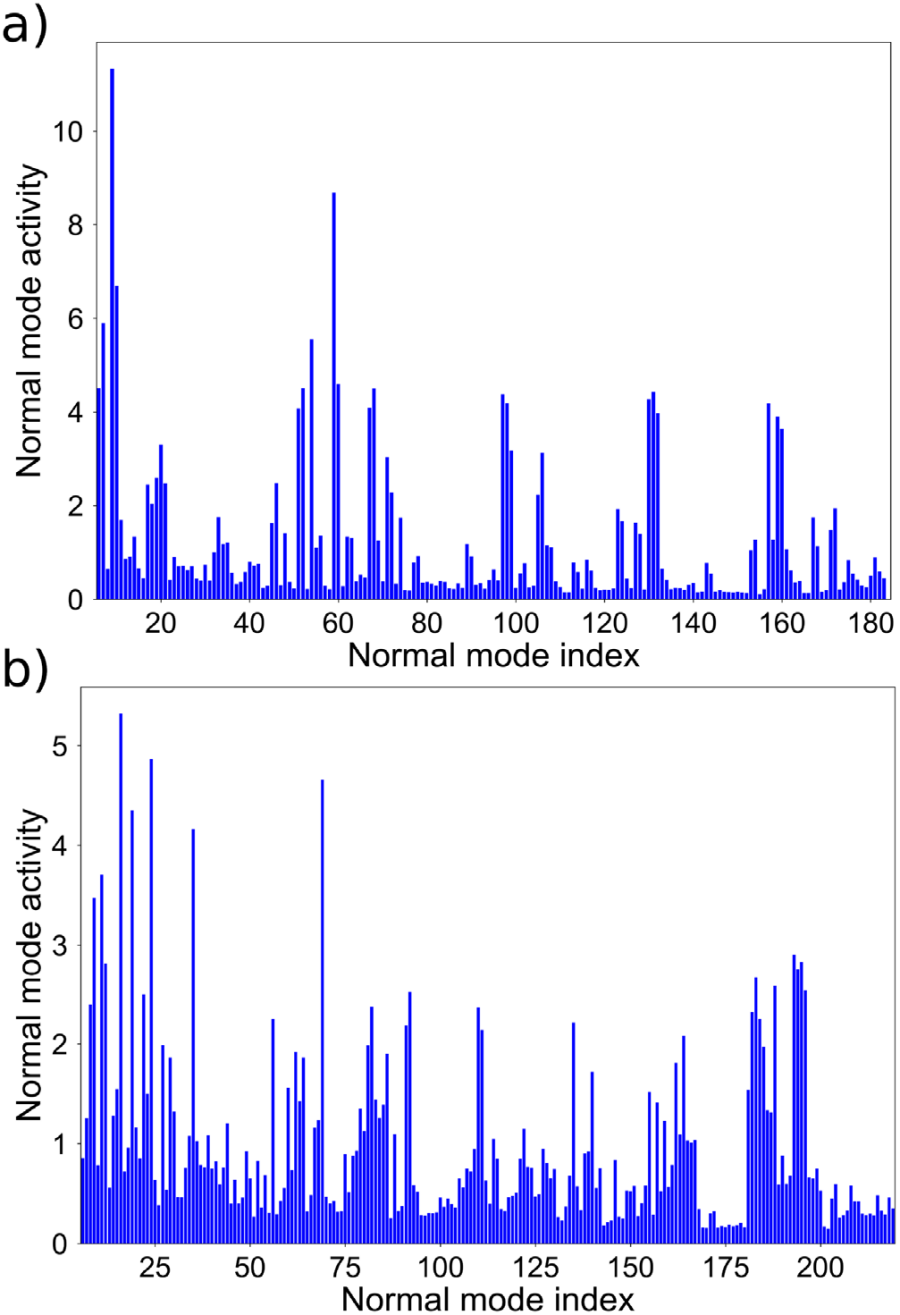
Normal‐mode activity along the TSH trajectories for (a) **Co1** and (b) **Co2**.

In order to investigate the role of vibrational motion further, the normal‐mode activity along the TSH trajectories for **Co1** and **Co2** according to Equation (4) has been analyzed (Figure 7). Overall, we observe that a few modes clearly stand out from an otherwise relatively constant background, indicating their high activity. In both cases, the modes are of antisymmetric Co–C stretching type, that is, they are LVC coupling modes. The three most active modes for **Co1** are $\nu_{9}=95$ cm^–1^, $\nu_{10}=95$ cm^–1^, and $\nu_{59}=678$ cm^–1^, whereas for **Co2** we find $\nu_{16}=153$ cm^–1^, $\nu_{24}=211$ cm^–1^, and $\nu_{69}=684$ cm^–1^. Overall, **Co1** has the two high $\sigma_{i}$ values, whereas in the case of **Co2**, more modes have appreciable activity values. This observation gives a more nuanced picture as compared with the discussion of the static coupling values in the previous section, both, however, agree in the more pronounced structural distortion of **Co2**.

**FIGURE 8:**
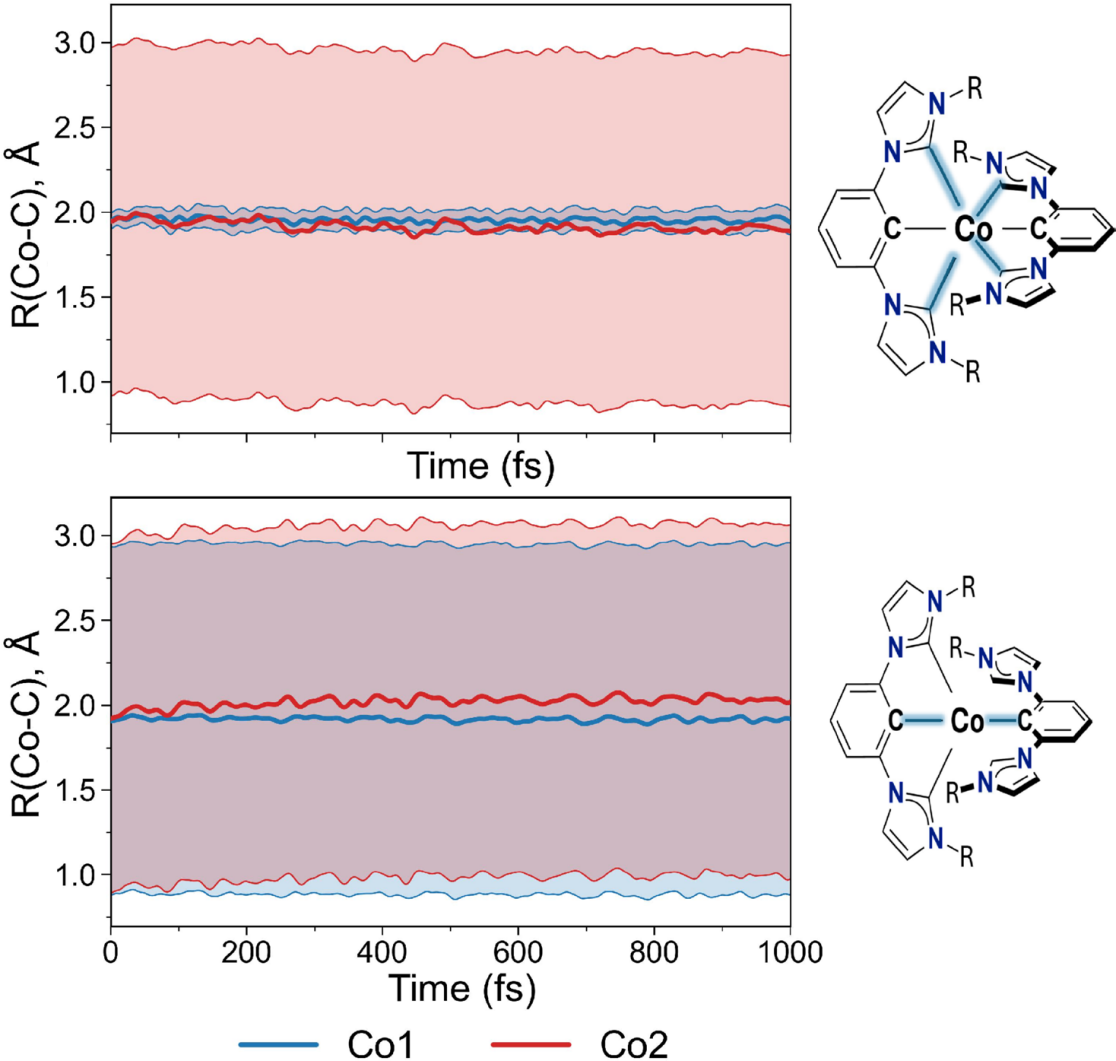
Time evolution of bond lengths for the Co—C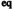 (upper panel) and Co—C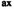 (lower panel) bonds, averaged for the same type of distances along the TSH trajectories. The corresponding RMSDs are shown as semitransparent shaded areas of the same colors.

A more global picture is provided by inspecting the temporal evolution of the Co–C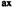 and Co—C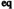 bond lengths and their associated RMSDs (Figure 8). Both complexes exhibit a small but gradual elongation of the Co—C bonds, indicative of structural relaxation within the excited singlet manifold. However, clear differences between the two systems emerge. In **Co1**, the average Co—C bond lengths remain relatively stable throughout the simulation, showing only moderate elongation and minor fluctuations. The RMSD quickly reaches a plateau after the initial relaxation phase, suggesting limited structural dispersion within the ensemble of trajectories and a comparatively rigid excited‐state PES along the metal–ligand stretching coordinates. In contrast, **Co2** displays more pronounced elongation of the Co—C bonds over time, particularly along the axial direction. This behavior is accompanied by a more noticeable increase in the RMSD, reflecting enhanced structural flexibility and a broader distribution of geometries sampled during the dynamics. This finding is in accord with the discussion in the previous section.

### 3.5 Minimum Energy Crossing Points

**FIGURE 9:**
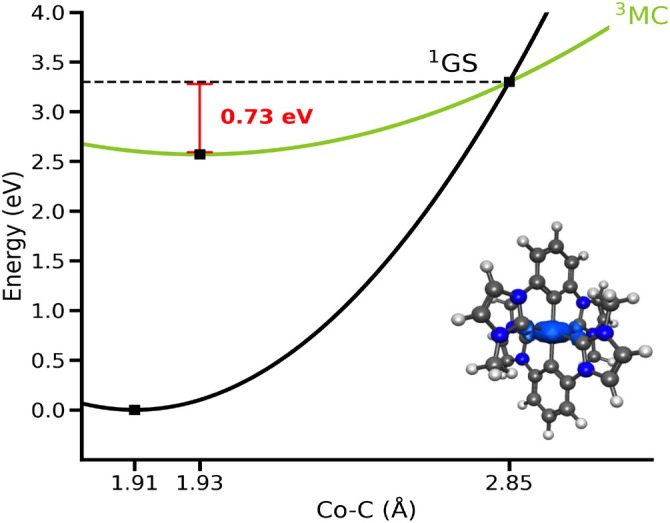
TD‐DFT calculated MECP between the 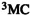 and 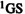 potential energy curves along the more elongated equatorial Co—C bond for **Co1** and spin density of the optimized triplet state.

Addressing the longest time scale observed in the experiment goes beyond the limitations of the LVC model. Therefore, geometry optimizations of the minimum energy crossing point (MECPs) between the ground state and the lowest ^3^MC state were performed for both **Co1** and **Co2**. In both complexes, the spin density of the triplet minima is localized on the metal center, as illustrated in Figure 9 for **Co1** (for **Co2** see Supporting Information, Figure S3), confirming their ^3^MC character. This lowest‐lying triplet state is likely the emissive state, consistent with experimental observations from low‐temperature emission and transient absorption spectroscopy [26].

Although the ^3^MC state is emissive at low temperatures, the experimentally observed quenching of emission at room temperature suggests the presence of a thermally accessible nonradiative decay pathway. The most probable mechanism is surface crossing from the ^3^MC state to the ground state via a MECP. Our calculations indicate that the MECP for **Co1** lies 0.73 eV above the ^3^MC minimum, while for **Co2** (see Supporting Information, Section S4) it is slightly higher at 0.89 eV (Figure 9). Analysis of the MECP geometries reveals pronounced distortion of the octahedral coordination environment in both complexes. In each case, one Co—C bond elongates significantly—to approximately 2.85 Å in **Co1** and 2.84 Å in **Co2**—while the remaining five Co–C bonds remain close to their equilibrium lengths (2.02 Å in **Co1** and 2.03 Å in **Co2**). Using the classification suggested in Ref. [61], the system corresponds to the sloped case, that is, there is a larger bond elongation in the MECP as compared to the ^3^MC minimum.

This selective bond elongation facilitates a rapid transition between the ^3^MC state and the ground state, as it effectively lowers the barrier for nonradiative decay. In general, population of antibonding orbitals in ^3^MC states induces significant metal–ligand bond elongation and structural distortion, leading to a reorganization of the potential energy surfaces and a shift of the corresponding minima relative to the ground‐ and ^3^MC‐state geometries. This distortion brings the system closer to MECP regions between electronic states, which are key points for nonradiative decay to the ground state [62]. Consistently, cyclometalated Ir(III) complexes [63] have been shown to undergo nonradiative decay through this sloped pathway, where the MECP geometry is characterized by further asymmetric elongation of the two Ir–N bonds (2.71 and 2.82 Å), indicating that these coordinates define access to the crossing region between the ^3^MC and the ground state and enabling efficient nonradiative decay.

These relatively low barriers indicate that nonradiative decay is thermally accessible at room temperature, explaining the loss of emission in both complexes under ambient conditions. At T=298 K, an Eyring‐type estimate based on the energy difference between the ^3^MC minimum and the MECP yields lifetimes of approximately 0.14 ns for **Co1** and 0.64 ns for **Co2**. This is in accord with the experimentally observed trend of slower relaxation in **Co2**. The calculated increase in lifetime by a factor of about 4.6 is in reasonable agreement with the experimentally observed increase from ~1 to ~2 ns [26].

We emphasize that this agreement should be interpreted cautiously, as the 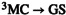 relaxation proceeds via a spin‐forbidden surface crossing and is therefore not strictly governed by conventional transition state theory. Nevertheless, the calculated MECP energetics successfully capture the relative trends in decay dynamics between the two complexes.

## 4 Conclusions

In this work, we employed TSH to investigate the ultrafast excited‐state relaxation mechanisms of two cyclometalated Co(III) complexes, bearing different alkyl groups (**Co1**: Methyl, **Co2**: Ethyl) at the nitrogen of the imidazole ligand. Previously, these complexes were studied experimentally in the context of low‐temperature triplet emission and room‐temperature excited‐state dynamics [26]. In fact, it was observed that even though the absorption spectra are rather similar, extracted time scales for dynamics are markedly different. Unraveling the substitution‐induced changes in the potential energy landscape that leads to this behavior has been the goal of the present study. The time constants extracted from the TSH simulations show excellent agreement with experimental particularly in capturing the initial picosecond decay ($\tau_{1}$) and the long‐lived ^3^MC state decay ($\tau_{3}$).

In both complexes, the dynamics is characterized by a rapid internal conversion within the singlet manifold leading to a dominant population of the two lowest singlet states within 200–300 fs. Intersystem crossing to the triplet manifold (^3^MLCT) occurs simultaneously. After about 200–300 fs, about 60% of the population is in ^3^MC states. This time scale is too fast to be compared with the experimental values of 1.2 ps (**Co1**) and 4.3 ps (**Co2**). Instead, the experimental $\tau_{1}$ is suggested to correspond to the time scale of intersystem crossing between the S_1_/S_2_ states and resonant triplet states. It is important to point out that this difference is found to be not due to the magnitude of the SOC, but rather a consequence of the different vibronic couplings. Compared to **Co1**, **Co2** features a broader range of vibronic couplings leading to larger structural distortions upon singlet manifold relaxation. In the spirit of the Markus theory, this feature effectively leads to a larger structural reorganization required for the singlet‐triplet transition. The longest time constant was found to be due to the intersystem crossing from the ^3^MC to the ground state. Here, the difference observed in the experiment could be rationalized in terms of the variation of the barrier defined by the Minimum Energy Crossing Point.

It should be noted that often the effect of ligand substituents on the excited‐state dynamics of transition‐metal photochemistry is discussed in terms of steric effects. Indeed, previous studies have demonstrated that steric modification of ligand frameworks can significantly influence excited‐state lifetimes by altering excited‐state structural relaxation pathways. However, the direction of this effect is highly system‐ and ligand‐dependent, with increased steric bulk either suppressing or promoting nonradiative decay, depending on how it perturbs the relevant region of the PES [64]. In heteroleptic Cu(I) complexes, for example, increased steric bulk typically suppresses excited‐state geometric relaxation and prolongs excited‐state lifetimes by limiting structural distortion [65]. At first glance, this appears to contrast with the present results. In the current system, however, replacing methyl groups in **Co1** with bulkier ethyl groups in **Co2** instead facilitates larger‐amplitude nuclear motion in the excited state. This finding suggests that, rather than rigidifying the structure, the increased steric encumbrance destabilizes the equilibrium geometry and promotes access to distorted configurations, contributing to the observed enhancement of $\tau_{1}$.

As far as the transition back to the ground state is concerned, the present **Co1** and **Co2** fall into a general scheme that has been observed in other transition‐metal complexes. Population of ^3^MC states is accompanied by significant metal–ligand bond elongation and structural distortion, reducing the energy gap to the ground state and enabling efficient nonradiative decay through accessible crossing regions [66]. This distortion‐mediated transition has been identified as a key nonradiative channel in first‐row transition‐metal complexes and cyclometalated Ir(III) species [63], where elongation of a metal–ligand bond at the MC minimum correlates with increased nonradiative decay rates. Such behavior is characteristic of a pseudo‐Jahn–Teller effect, in which asymmetrical relaxation along nuclear coordinates facilitates efficient nonadiabatic transitions. Consequently, this structural rearrangement provides a mechanistic explanation for the rapid quenching of emission at room temperature [61].

## Funding

This work is funded by the Deutsche Forschungsgemeinschaft (DFG) through projects KU 952/12‐2 (O.K.) and the Research Training Group GRK 2943 ‘‘Spectroscopic Tools for Challenging Reduction Reactions: Catalytic Coupling of CO2’’, project no. 507189291 (O.S.B.) as well as by the Leibniz Association through the Leibniz Competition (O.S.B.).

## Supporting information


**Data S1:** Tuning of the LC‐BLYP functional for DFT calculations.
**S2:** Calculated structural properties of **Co1** and **Co2**.
**S3:** ^3^MLCT‐^3^MC crossing.
**S4:** Minimum energy crossing points for **Co2**.
**S5:** Analysis of spin‐orbit couplings within a two‐state model.
**S6:** Analysis of population beatings.
**Table S1:** Selected structural parameters and relative energies for **Co1** and **Co2** for the ^1^GS, ^3^MC, ^3^MLCT, and ^3^MC states.
**Figure S1:** Spin densities of the lowest ^3^MLCT and ^5^MC states of **Co1** and **Co2** at their relaxed geometries (isovalue = 0.02). Panels (a) and (b) correspond to the ^3^MLCT and ^5^MC states of **Co1**, respectively, while panels (c) and (d) correspond to the ^3^MLCT and ^5^MC states of **Co2**, respectively.
**Figure S2:** PESs of **Co1** (a) and **Co2** (b) along the lowest ^3^MLCT and ^3^MC states obtained by unrestricted DFT calculations. Note that the minima correspond to the separately optimized states. They are connected along the Co—C_eq_ bond distance, keeping all other coordinates frozen.
**Figure S3:** TD‐DFT calculated MECP between the ^3^MC and ^1^GS potential energy curves along the more elongated equatorial Co—C bond for **Co2** and spin density of the optimized triplet state.
**Figure S4:** Transfer time T = π/Ω for all pairs of S_1_/S_2_ and triplet states for the two complexes (note the log scale).
**Figure S5:** Transfer fraction 4|V_SOC_|^2^/(¯hΩ)^2^ for all pairs of S_1_/S_2_ and triplet states for the two complexes (note the log scale).
**Figure S6:** Analysis of population beating observed in Fig. 5. Panels (a,b) and (c,d) show the population dynamics and its Fourier amplitude spectrum for **Co1** and **Co2**, respectively.
**Figure S7:** Population dynamics for a simulation including states S_1_ and S_2_ only. Panels (a,b) and (c,d) show the population dynamics and its Fourier amplitude spectrum for **Co1** and **Co2**, respectively.

## Data Availability

Data available from the authors upon reasonable request.
