# Supplementary material for: How Structural Subtleties Modulate Excited‐State Lifetimes in Cyclometalated Cobalt(III) Complexes: Nonadiabatic Molecular Dynamics Study
Source: J Comput Chem. 2026 Jul 20;47(20):e70453. doi: 10.1002/jcc.70453 (PMC13385668; doi:10.1002/jcc.70453)
Supplement: Supplementary file 1 — Data S1: Tuning of the LC‐BLYP functional for DFT calculations. S2: Calculated structural properties of Co1 and Co2. S3: 3MLCT‐3MC crossing. S4: Minimum energy crossing points for Co2. S5: Analysis of spin‐orbit couplings within a two‐state model. S6: Analysis of population beatings. Table S1: Selected structural parameters and relative energies for Co1 and Co2 for the 1GS, 3MC, 3MLCT, and 3MC states. Figure S1: Spin densities of the lowest 3MLCT and 5MC states of Co1 and Co2 at their relaxed geometries (isovalue = 0.02). Panels (a) and (b) correspond to the 3MLCT and 5MC states of Co1, respectively, while panels (c) and (d) correspond to the 3MLCT and 5MC states of Co2, respectively. Figure S2: PESs of Co1 (a) and Co2 (b) along the lowest 3MLCT and 3MC states obtained by unrestricted DFT calculations. Note that the minima correspond to the separately optimized states. They are connected along the Co—Ceq bond distance, keeping all other coordinates frozen. Figure S3: TD‐DFT calculated MECP between the 3MC and 1GS potential energy curves along the more elongated equatorial Co—C bond for Co2 and spin density of the optimized triplet state. Figure S4: Transfer time T = π/Ω for all pairs of S1/S2 and triplet states for the two complexes (note the log scale). Figure S5: Transfer fraction 4|VSOC|2/(¯hΩ)2 for all pairs of S1/S2 and triplet states for the two complexes (note the log scale). Figure S6: Analysis of population beating observed in Fig. 5. Panels (a,b) and (c,d) show the population dynamics and its Fourier amplitude spectrum for Co1 and Co2, respectively. Figure S7: Population dynamics for a simulation including states S1 and S2 only. Panels (a,b) and (c,d) show the population dynamics and its Fourier amplitude spectrum for Co1 and Co2, respectively. [file JCC-47-0-s001.pdf]

Supporting Information: How Structural Subtleties Modulate Excited-State Lifetimes in Cyclometalated Cobalt(III) Complexes: Nonadiabatic Molecular Dynamics Study

Hamada Rezk and Oliver Kühn*
Institute of Physics, University of Rostock,
Albert-Einstein-Str. 23-24, D-18059, Rostock, Germany

Olga S. Bokareva
Leibniz Institute for Catalysis (LIKAT),
Albert-Einstein-Str. 29A, D-18059, Rostock, Germany and
Institute of Chemistry, University of Rostock,
Albert-Einstein-Str. 27A, D-18059, Rostock, Germany

CONTENTS

S1: Tuning of the LC-BLYP functional for DFT calculations

S2: Calculated structural properties of **Co1** and **Co2**

S3: ^{3}MLCT - ^{3}MC Crossing

S4: Minimum Energy Crossing Points for **Co2**

S5: Analysis of Spin-orbit Couplings Within a Two-state Model

S6: Analysis of Population Beatings

* oliver.kuehn@uni-rostock.de

S1. TUNING OF THE LC-BLYP FUNCTIONAL FOR DFT CALCULATIONS

The functional $J^*(\alpha, \omega)$ is defined as:[1]

$$J^*(\alpha,\omega) = \sqrt{J_0^2 + J_1^2},$$
$$\text{where} \quad J_0 = |\epsilon_{\text{HOMO}}(N) + \text{IP}(N)|\,,$$
$$J_1 = |\epsilon_{\text{HOMO}}(N+1) + \text{EA}(N)|\,.$$

showing the deviation from Koopman's theorem for ionization potential (IP) and electron affinity (EA) for the system with N electrons. As a result of tuning, the parameters α=0.0 and ω=0.15 Bohr^{-1} for **Co2**. The same parameters are used for **Co1**.

S2. CALCULATED STRUCTURAL PROPERTIES OF CO1 AND CO2

The character of the ^{3}MLCT and ^{5}MC excited states at their respective optimized geometries was verified by spin-density analysis, as illustrated in Figure S1.

For each complex, the structural parameters in Table S1 are averaged over axial and equatorial sites. In the ^1GS, Co–C$_{ax}$ bonds are slightly shorter than Co–C$_{eq}$, with nearly linear C$_{ax}$–Co–C$_{ax}$ and more compressed equatorial angles, consistent with a distorted octahedral geometry optimized for a closed-shell configuration.

TABLE S1: Selected structural parameters and relative energies for **Co1** and **Co2** for the ^{1}GS, ^{3}MC, ^{3}MLCT, and ^{3}MC states.

System	State	ΔE (eV)	Co–C_{ax} (Å)	Co–C_{eq} (Å)	C_{ax}–Co–C_{ax} (°)	C_{eq}–Co–C_{eq} (°)
**Co1**	^{1}GS	0.00	1.91	1.93	179.99	157.65
	^{3}MC	2.67	1.93	2.12	179.89	156.06
	^{3}MLCT	3.27	1.93	1.95	179.65	159.80
	^{5}MC	4.84	2.09	2.32	160.18	148.66
**Co2**	^{1}GS	0.00	1.92	1.94	179.29	157.84
	^{3}MC	2.64	1.93	2.10	179.68	155.34
	^{3}MLCT	3.26	1.93	1.96	179.57	159.88
	^{5}MC	4.80	2.08	2.14	159.74	148.89

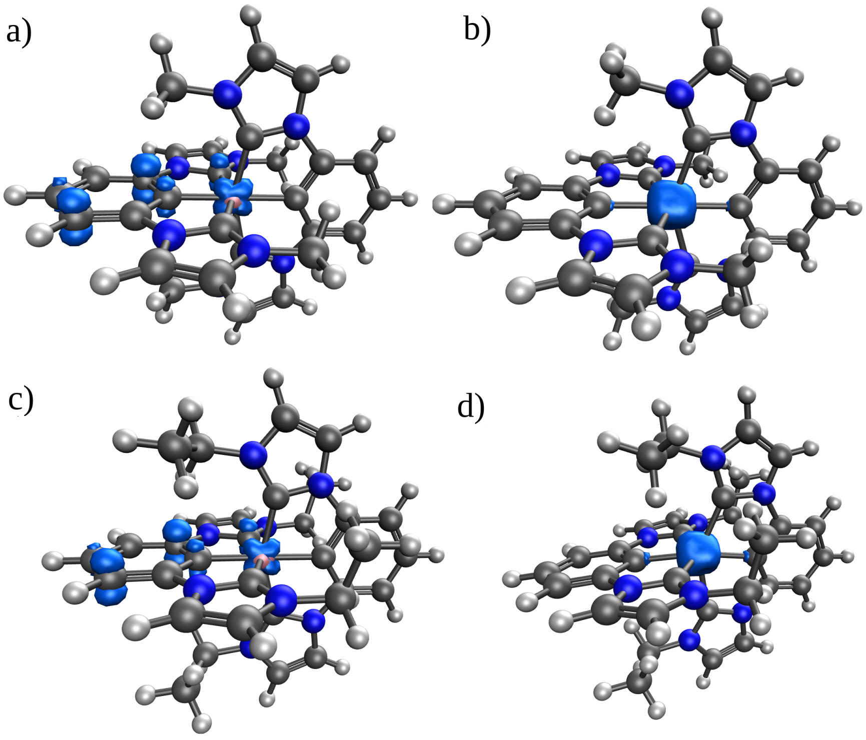

FIG. S1: Spin densities of the lowest ^{3}MLCT and ^{5}MC states of **Co1** and **Co2** at their relaxed geometries (isovalue = 0.02). Panels (a) and (b) correspond to the ^{3}MLCT and ^{5}MC states of **Co1**, respectively, while panels (c) and (d) correspond to the ^{3}MLCT and ^{5}MC states of **Co2**, respectively.

Upon excitation, the ^{3}MC and ^{3}MLCT states show contrasting behavior. The ^{3}MC state exhibits modest axial elongation but a more pronounced increase in equatorial Co–C distances, while maintaining an almost linear axial angle. This indicates preferential weakening of equatorial metal–ligand bonds due to population of antibonding metal-centered *d* orbitals. In contrast, the ^{3}MLCT state remains close to the ^{1}GS geometry, with only minor changes in bond lengths and angles, consistent with a more delocalized excitation and limited structural reorganization.

The ^{5}MC state shows a much larger distortion, with substantial elongation of both axial and equatorial Co–C bonds and a marked deviation from linearity in the axial angle, reflecting strong weakening of metal–ligand interactions and a highly distorted coordination sphere. This state is also significantly higher in energy than the triplet states (Table S1), indicating reduced accessibility.

Overall, the relatively low energy and moderate distortion of the ^{3}MC state suggest it as

the primary relaxation channel. The ^{3}MLCT state, being structurally close to the ^{1}GS, is less involved in large-amplitude nuclear motion, while the high-energy, strongly distorted ^{5}MC state is unlikely to contribute significantly to non-radiative decay. These trends agree with established models of transition-metal photophysics, where low-lying ^{3}MC states dominate deactivation pathways, as observed in related iron systems [2].

S3. ^{3}MLCT - ^{3}MC CROSSING

To rationalize the experimentally observed τ_2 time in view of a possible ^{3}MLCT - ^{3}MC transition, we examined PES scans along the Co–C$_{eq}$ bond for the relevant ^{3}MLCT (T_4) and ^{3}MC (T_1) states. As can be seen from Fig. S2 in both **Co1** and **Co2**, the two surfaces intersect at approximately 2.03 Å essentially barrierless. In view of the TSH results, this finding suggests that the transient population of the ^{3}MLCT states comes along with a relaxation down to the lowest ^{3}MLCT state, which rapidly converts into ^{3}MC populations. Overall, within the present model, there is no indication of a large difference in dynamics between **Co1** and **Co2** that could give rise to the rather different τ_2 times.

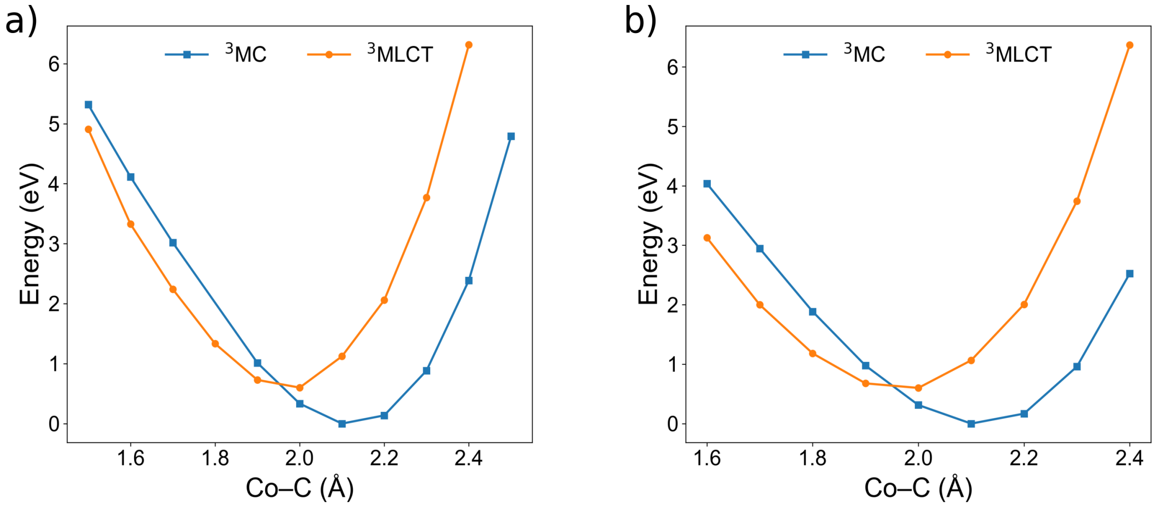

FIG. S2: PESs of **Co1** (a) and **Co2** (b) along the lowest ^{3}MLCT and ^{3}MC states obtained by unrestricted DFT calculations. Note that the minima correspond to the separately optimized states. They are connected along the Co-C$_{eq}$ bond distance, keeping all other coordinates frozen.

S4. MINIMUM ENERGY CROSSING POINTS FOR CO2

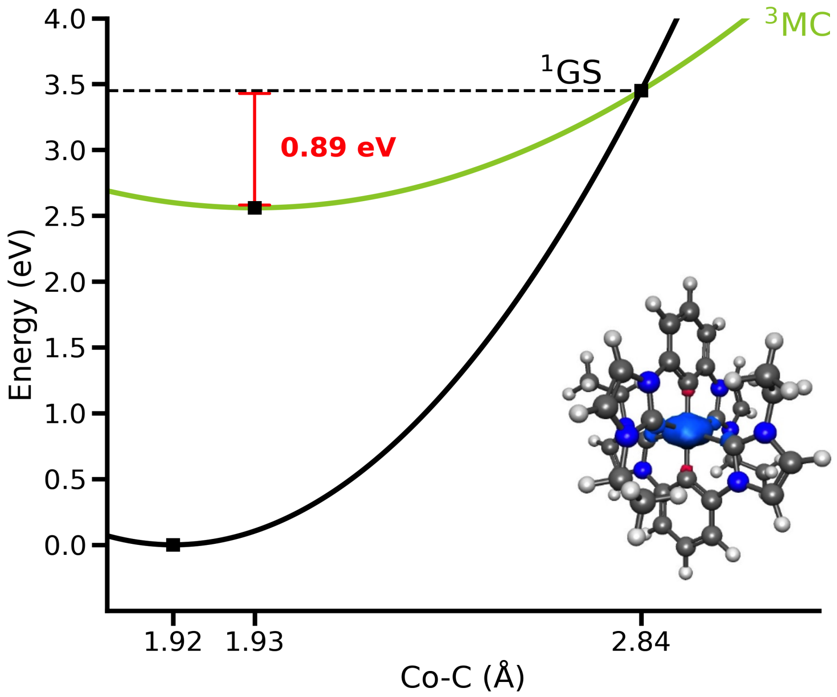

FIG. S3: TD-DFT calculated MECP between the ^{3}MC and ^{1}GS potential energy curves along the more elongated equatorial Co-C bond for **Co2** and spin density of the optimized triplet state.

S5. ANALYSIS OF SPIN-ORBIT COUPLINGS WITHIN A TWO-STATE MODEL

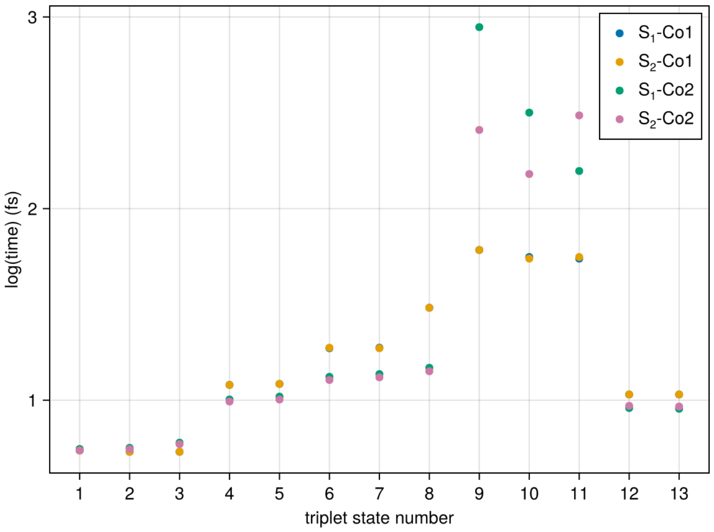

FIG. S4: Transfer time $T = \pi/\Omega$ for all pairs of S_1/S_2 and triplet states for the two complexes (note the log scale).

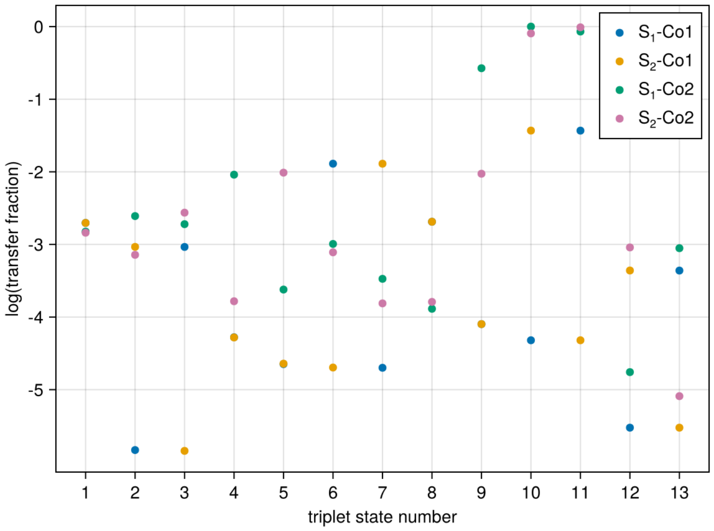

FIG. S5: Transfer fraction $4|V_{\mathrm{SOC}}|^2/(\hbar\Omega)^2$ for all pairs of S_1/S_2 and triplet states for the two complexes (note the log scale).

Treating each pair of S-T states as a coupled two-level system, an initially prepared singlet state will perform periodic oscillations with the transition amplitude[3]

$$P_{\mathrm{S}\to\mathrm{T}} = \frac{4|V_{\mathrm{SOC}}|^2}{(\hbar\Omega)^2}\sin^2\left(\frac{\Omega t}{2}\right)$$

with $\hbar\Omega = \sqrt{(E_\mathrm{S} - E_\mathrm{T})^2 + 4|V_{\mathrm{SOC}}|^2}$. Hence, at $T = \pi/\Omega$ the triplet state is populated by an amount of $4|V_{\mathrm{SOC}}|^2/(\hbar\Omega)^2$, depending on the detuning and the coupling.

In Fig. S4, we show $T = \pi/\Omega$ for all pairs of $\mathrm{S}_1/\mathrm{S}_2$ and triplet states for the two complexes. Fig. S5 shows the respective transfer fraction $4|V_{\mathrm{SOC}}|^2/(\hbar\Omega)^2$.

S6. ANALYSIS OF POPULATION BEATINGS

In what follows, we focus on the population beating between the two lowest excited singlet states shown in Fig. 5. The beating manifests as out-of-phase oscillations of the two diabatic populations. This anti-correlation is a direct signature of coherent population transfer between the electronic states mediated by the off-diagonal vibronic coupling. To analyze the beating, the oscillatory part of the population starting from 200 fs has been Fourier transformed. The results for the two complexes are shown in Fig. S6. Due to the limited spectral resolution and the relatively dense normal mode spectrum in the ranges of interest, the following discussion will focus on spectral ranges instead of specific frequencies.

For **Co1** dominant frequencies are in the range between 1200 and 1400 cm^{-1}, whereas for **Co2** the range is between 400 and 500 cm^{-1}. In these ranges, modes generally have small κ values, indicating only small relative shifts between the PES. Further λ values are small as well, making the coupling term $\lambda q(t)$ a perturbation, whose magnitude is determined by

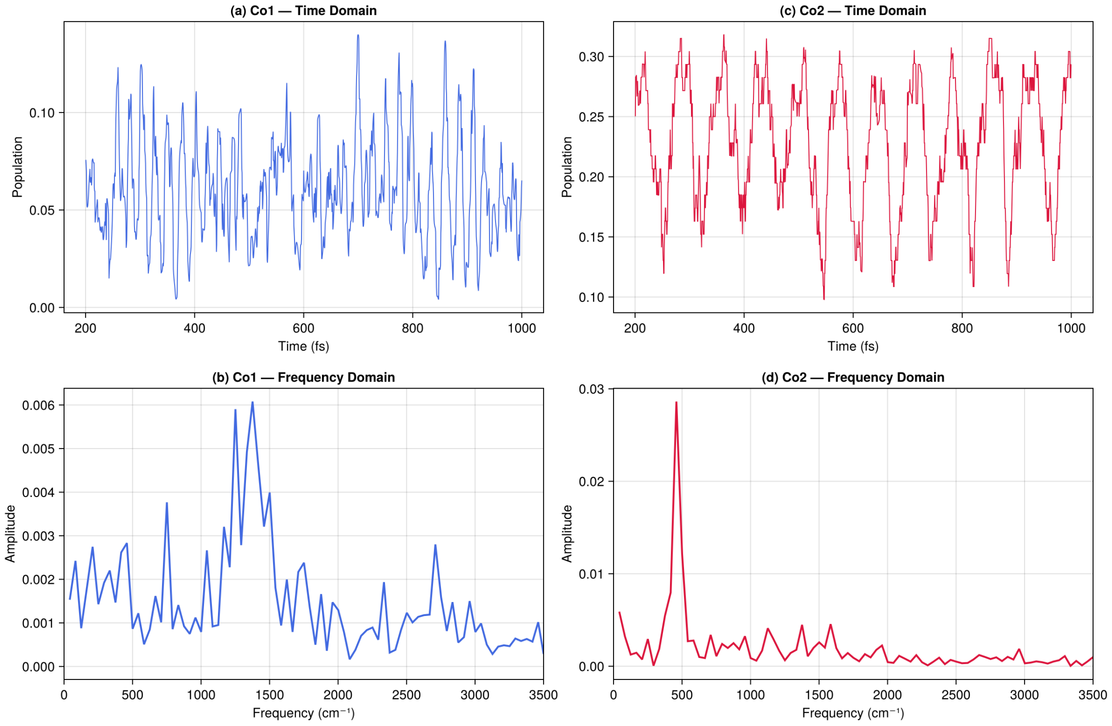

FIG. S6: Analysis of population beating observed in Fig. 5. Panels (a,b) and (c,d) show the population dynamics and its Fourier amplitude spectrum for **Co1** and **Co2**, respectively.

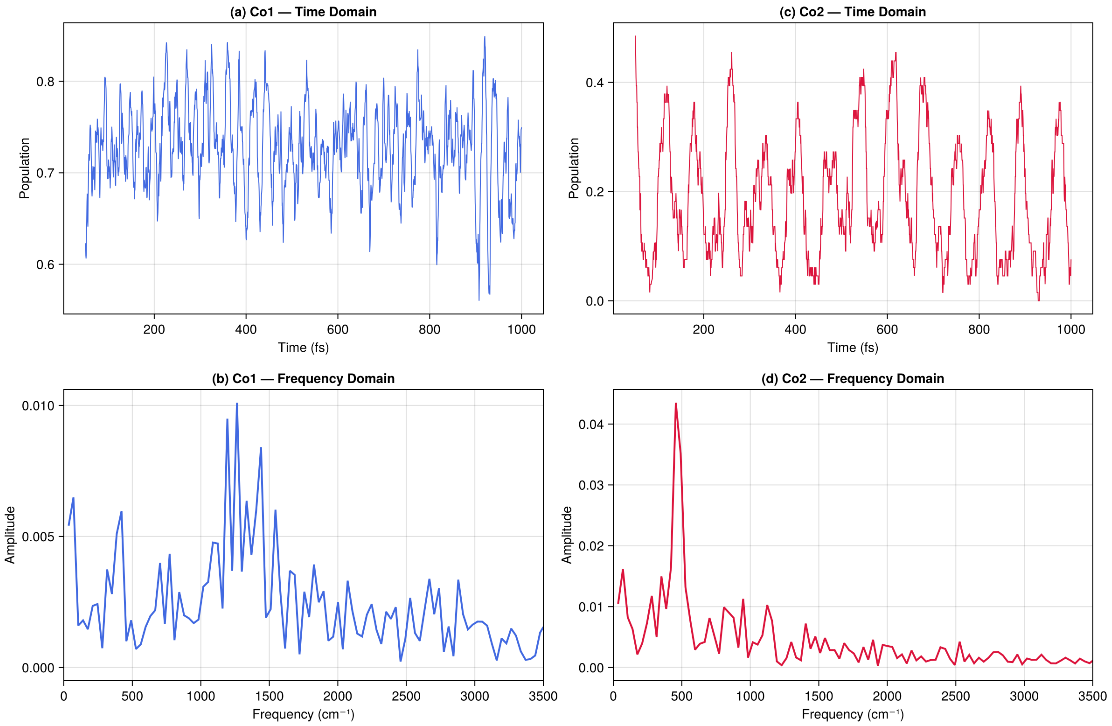

FIG. S7: Population dynamics for a simulation including states S_1 and S_2 only. Panels (a,b) and (c,d) show the population dynamics and its Fourier amplitude spectrum for **Co1** and **Co2**, respectively.

initial preparation (Wigner sampling) and, in principle, the initial relaxation from higher excited states. To exclude the second possibility, we have performed TSH simulations including the S_1 and S_2 states only. The result is shown in Fig. S7. Overall, the range of dominant modes compares well to the full model, ruling out relaxation as a trigger for the observed beating.

In principle, it is surprising to see the effects of specific modes for a system having about 200 vibrational degrees of freedom. Hence, one might ask the question whether this could be an artifact of TSH. For instance, 200 production trajectories will impossibly sample the about 400-dimensional phase space. On the other hand, we checked that for the given 200 trajectories, there is little variation in the beating if different numbers of trajectory is included in the averaging. Of course, this doesn't rule out that the situation will change if substantially more trajectories would be included. Further, it is known that TSH overemphasizes coherence. However, we have accounted for this fact by working with a decoherence correction. Finally, to explore this point further, we have performed preliminary all-quantum

simulations for **Co1** [4], which also showed a population beating. Here, the dominant contribution is around 500 cm^{-1}, and the amplitudes in the 1200 to 1400 $cm^{-1}$are not as prominent as in the TSH results. Thus, we conclude that for the given LVC Hamiltonian, beating between the lowest excited diabatic singlet state populations is a real effect. However, the present TSH simulations likely overemphasize certain modes.

[1] J. P. Zobel, A. Kruse, O. Baig, S. Lochbrunner, S. I. Bokarev, O. Kühn, L. González, and O. S. Bokareva, Chem. Sci. **14**, 1491 (2023).

[2] L. A. Fredin, M. Papai, E. Rozsalyi, G. Vankó, K. Warnmark, V. Sundstrom, and P. Persson, J. Phys. Chem. Lett. **5**, 2066 (2014).

[3] V. May and O. Kühn, *Charge and Energy Transfer Dynamics in Molecular Systems*, 4th ed. (Wiley-VCH, Weinheim, 2023).

[4] F. Bogdain, S. Mai, L. González, and O. Kühn, Phys. Chem. Chem. Phys. **27**, 15609 (2025).
